# Effectiveness of virtual teaching of diagnostic and interventional imaging fundamentals to Egyptian medical students: an analytical cross-sectional study

**DOI:** 10.1186/s43055-021-00688-7

**Published:** 2022-01-10

**Authors:** Ahmed S. Negm, Ahmed Elhatw, Mohamed Badawy, Meredith L. Gioe, Sana Khan, Mahmoud F. Hammad, Nada Shalaby, Ferial Choucair, Mariam Ahmed Saad, Amany Elfeel, Mariam Elbatal, Florentino Saenz, Mostafa Ahmed Shehata, Parth Patel, Muhammad Aly, Islam Khalifa, Serageldin Kamel, Yara ElHefnawi, Mohamed Ismael Fahmy, Scott Rohren, Mohamed Yasser Hussein, Ahmed Elsaiey, Owiss Zitoun, Khaled M. Elsayes

**Affiliations:** 1 Mayo Clinic, Rochester, MN USA; 2 National Cancer Institute, Cairo University, Cairo, Egypt; 3 University of Texas MD Anderson Cancer Center, Houston, TX USA; 4 Louisiana State University Health Sciences Center, New Orleans, LA USA; 5 University of Houston, Houston, TX USA; 6 National Liver Institute, Menoufia University, Menoufia, Egypt; 7 Faculty of Medicine, Alexandria University, Alexandria, Egypt; 8 Faculty of Medicine, Ain Shams University, Cairo, Egypt; 9 University of Texas Health San Antonio, San Antonio, TX USA; 10 McGovern Medical School at UTHealth, Houston, TX USA; 11 University of Texas Medical Branch at Galveston, Galveston, TX USA; 12 Kasr Al-Ainy Medical School, Cairo University, Cairo, Egypt; 13 Faculty of Medicine, Mansoura University, Mansoura, Egypt; 14 Baylor College of Medicine, Houston, TX USA; 15 Faculty of Medicine, The Memorial Souad Kafafi University Hospital, Misr University for Science and Technology, Giza, Egypt; 16 Houston Methodist Hospital, Houston, TX USA

**Keywords:** Radiology, Education, Undergraduate, Online course, Medical student

## Abstract

**Background:**

There is a worldwide deficit in teaching and training in the field of radiology for undergraduate medical students. This educational gap is prominent in many medical schools as most radiology curricula are a part of other specialty trainings, usually provided by non-radiologists. After COVID-19 pandemic, there was an increased trend in online education. However, questions have been raised about the efficacy and acceptance of online education. We developed a course on the principles of radiology and medical imaging basics to target Egyptian medical students. We then assessed the impact of these educational videos through several online surveys. Our "The Principles of Radiology Online Course" was delivered to students at various Egyptian medical schools; it was a prerecorded series composed of nine sessions, and each session followed the sequence of a pre-test, video, and post-test. There was a final survey to assess the overall feedback. Finally, we analyzed the results to give insight onto how teaching radiology through online lectures can help build better physicians.

**Results:**

Among various medical schools around Egypt, 1396 Egyptian medical students joined this cohort. Cohort population percentage was 56% female and 44% male. Ninety-eight percent of the students agreed that this program increased their understanding of radiology. Eighty-four percent of the students found the platform friendly and easy to use. Seventy-nine percent found these webinars were more convenient compared to in-person education. Statistical significance (*p-*value < 0.05) was achieved in all sessions after comparing students’ pre and post-test scores, and in students’ confidence and knowledge level before and after the course.

**Conclusions:**

Radiology is an underrepresented subject for a lot of medical students. Online radiology webinars have proven to be a promising method of teaching medical students key medical imaging concepts. An online course of radiology basics and principles can help improve a medical student’s knowledge and enhance overall future patient care.

## Introduction

COVID-19 pandemic has affected medical education in Egypt, as well as other countries [1, 2]. This led to shifting to online medical education platforms [3, 4]. The literature has assessed the impact of COVID-19 on medical education and the benefits of virtual classrooms [5–7]. Radiology was among the disciplines involved in the online medical education era [8, 9].

Students spent loads of time and effort in medical education to prepare themselves for future clinical practice. Despite the increasing role of imaging in the medical field, radiology education is not receiving the required attention [10]. Multiple studies have shown that medical students worldwide are lacking the basics necessary for imaging indication and interpretation [10–12]. Recent literature has shown that medical students are aware of the importance of radiology education but unpleased with their undergraduate radiology curriculum [13]. The worldwide lack of basic undergraduate radiology knowledge has resulted in avoidable outcomes, such as difficulty interpreting basic imaging findings or ordering appropriate imaging by interns in their early years of clinical practice (internal medicine, surgery, pediatrics) [12]. Therefore, we think that radiology undergraduate education should be delivered by radiology specialists instead of integrating it among other specialties delivered by non-radiologists [11].

To the best of our knowledge, no study has addressed the lack of undergraduate radiology education in Egypt specifically; all conducted ones enlisted Egypt among other countries [14, 15]. Our study could be the first to address this topic. Here, we assess the efficacy of our online ‘principles of radiology’ course for Egyptian medical students to give an example to reimplement it on a larger global scale.

## Methods

We took informed consent from the participating students to use their survey answers and analysis for research purposes. An analytical cross-sectional study was conducted in our course period during February–May 2021.

### Design and development

Editorial board of our radiology academy designed an online radiology principles course to Egyptian medical students. This course aimed to boost students’ knowledge with a specialized radiology education.

The course was designed and reviewed by our editorial board of radiology professors—from various renowned medical schools and universities among the United States and Canada, and who have many years of experience in radiology teaching. The course aimed to elaborate the principles of radiology in a series of nine pre-recorded lectures, each around an hour long. The lectures focused on different imaging modalities and discussed radiation and contrast safety with their respective topics. The learning objective for Phase I was to set a startup for all students regarding basic radiological imaging. Table 1 lists the titles of the 9 lectures taught over a designated period of 1 month (February 2021). Phase II comprised of the Multidisciplinary Teaching of Radiology, in which a specialized radiologist and another specialist (i.e., surgeon) engaged in a live classroom where they integrated multiple diseases, both clinically and radiologically. Phase II live sessions were held on a biweekly basis in a period of approximately 2.5 months (March–May 2021) [16].

**Table 1:** List of the topics discussed throughout the course

Session	Number of students completed both tests	Pre-test mean score (SD)	Post-test mean score (SD)	*p* value
*Phase I*				
Principles of X-ray	2723	3.70 (1.87)	7.50 (2.07)	< 0.001
Clinical correlation of the chest radiographs	2080	5.46 (2.13)	8.29 (2.00)	< 0.001
Principles of CT	1787	4.64 (2.20)	7.70 (2.00)	< 0.001
Principles of MRI	1616	4.10 (1.83)	7.66 (2.32)	< 0.001
Principles of ultrasonography	1571	5.73 (2.15)	8.18 (2.04)	< 0.001
Principles of contrast agents	1538	5.10 (1.64)	6.87 (1.85)	< 0.001
Principles of PET and nuclear medicine	1457	4.90 (2.05)	6.94 (2.06)	< 0.001
Introduction to interventional radiology	1429	6.87 (2.53)	8.44 (2.03	< 0.001
Journey from roentgen to artificial intelligence	1535	2.70 (1.60)	4.47 (2.18)	< 0.001
*Phase II*				
Child abuse	735	4.73 (1.43)	6.91 (1.78)	< 0.001
Bone fractures	758	4.20 (1.53)	7.53 (2.03)	< 0.001
Breast	794	5.08 (1.87)	7.25 (2.10)	< 0.001
Pneumonia	682	4.89 (1.64)	7.36 (2.01)	< 0.001
Flank pain	703	3.31 (1.82)	6.52 (2.72)	< 0.001

Throughout the course, each lecture had a pre-test–post-test design. An end of course survey was filled to measure the impact and feedback of the overall experience of students.

### Recruitment of ambassadors

In nearly two weeks, we formed a team of 36 medical student ambassadors from different medical schools to cover most of Egypt (e.g., Cairo, Ain Shams, Alexandria, Al-Azhar, Benha, New Giza, Zagazig, Menoufia, Fayoum, Sohag, Assiut, Aswan). Each medical school had about 1 to 3 ambassadors. An ambassador had volunteered to join our team and represent their medical school after we reached out students’ Facebook groups (Facebook, Inc., Menlo Park, CA) for recruitment and elaborated our course objectives. Apart from participating in online social media and students’ extracurricular activities, there were no certain ambassador selection criteria. Ambassadors’ role was to use social media platforms to announce for course enrollment to their colleagues at their medical school. The main platforms used were medical school Facebook groups and Twitter (Twitter, Inc., San Francisco, CA), which had the highest accessibility and reach rates among medical students in Egypt. The ambassadors designed an online campaign, shared it along with registration forms, and answered students’ questions. The main channel of communication between registered students and ambassadors was through e-mails and WhatsApp groups (WhatsApp, Inc., Menlo Park, CA). This might explain that 78% of students heard about our course from social media.

### Enrollment of candidates

We aimed to benefit the largest number possible of students; most candidates were either a student or a house officer in their final year in an Egyptian medical school. The number of registered candidates (~ 10,000) exceeded the estimated rates, and therefore, a modification was formed to their selection criteria. Only students who finished phase I (the prerecorded lectures of Radiology Principles) within a timeframe of approximately 1 month were eligible to attend phase II. From 10,000 students who applied to phase I, only 1364 passed on to phase II, in which they were split into two groups. Phase II live sessions were repeated twice for each group.

### Tools

The prerecorded lectures were sent via email to the enrolled students for them to access online at their own pace within a convenient period of approximately 1 month. The live sessions were streamed as video conferences on Zoom (Zoom Video Communications, San Jose, CA); each was an hour long and had a 15-min question and answer (Q&A) session in the end. Our team members coordinated the online rooms to facilitate screen-sharing as doctors combined their presentations. A chat box was utilized to share the pre-test and post-test links and organize the designated Q&A portion at the end of each lecture. This planned management was purposed to mimic the face-to-face educational experience. Lecture handouts were made and provided to the students via e-mail to enhance their learning experience.

### Assessment

Pre-test and post-test drafts were constructed by medical students who previously attended the course trials in the USA and Canada. Each lecturer reviewed the test drafts and modified questions as needed to tailor the tests to their lectures content. Pre- and post-tests consisted of 10 multiple choice question per lecture. They were presented using Qualtrics software (Qualtrics, Provo, UT, USA). Data from the test scores were calculated; the pre-test and post-test scores for each student were matched and analyzed using a Student’s paired *t*-test with a *p*-value of less than 0.05.

The end-of-course survey was conducted through Survey monkey (SurveyMonkey Inc., California, USA). This survey collected demographic data about the student, the status of radiology education in their medical schools, and student ratings of each given lecture. It also heavily focused on the student’s status regarding their interest, understanding, and confidence in the field of radiology before and after completing this course. Subjective survey data were analyzed to measure statistical significance.

## Results

### Statistical analysis

Data of pre- and post-test scores were analyzed using a paired *t*-test on Microsoft Excel software (Microsoft, Redmond, Washington); a *p*-value of less than 0.05 was used as a measure of statistical significance between pre- and post-test score differences (Table 1). Only scores of students who completed both the pre- and post-tests were included in the analysis. After completion of the whole course (phases I and II), students completed a subjective evaluation survey on a four-point Likert scale to assess their confidence level of the learned radiology skills. MATLAB (The MathWorks, Natick, Massachusetts) then was used to perform the Wilcoxon signed-rank test (with a one-tailed hypothesis) to compare between confidence levels before and after the course, with a *p*-value of less than 0.05 (Table 2).

**Table 2:** Statistical analysis of students’ confidence level before and after the course (Phases I and II)

Confidence level	Weighted average before the session	Weighted average after the session	*p* value
I am familiar with best-use radiology practices	1.91	3.02	< 0.001
I am comfortable interpreting radiographs	1.84	2.94	< 0.001
I understand safety in radiology	2	3.12	< 0.001
I am able to identify gross abnormalities on imaging	2.17	3.16	< 0.001
I am familiar with how imaging is used as a diagnostic tool	2.28	3.26	< 0.001
I am familiar with the different imaging modalities and when to use them	2.12	3.23	< 0.001

### Study results

More than 10 thousand medical students initially enrolled in the program by completing an online sign-up interest form. An average of 1748 attendees completed both the pre-test and post-test for each session. A total of 1396 students answered the end-of-course survey with 1170 (83.81%), stating that they attended the whole 9 sessions. Gender distribution was 605 (43.3%) males, 787 (56.3%) females, and 4 (0.29%) preferred not to answer. Pre- and post-test scores of all course sessions were recorded, with statistical significance in all sessions (*p*-value < 0.001) (Table 1).

A total of nearly 70% were students among first six years of medical schools, while the remainder were either house officers or non-radiology staff. Attendees were enrolled at medical schools across 9 different countries with 99.14% of the respondents studying medicine in Egypt. When asked if a radiology clerkship is required at their medical schools, nearly half (45.2%) stated that a radiology clerkship is not required at their medical schools.

**Fig. 1:**
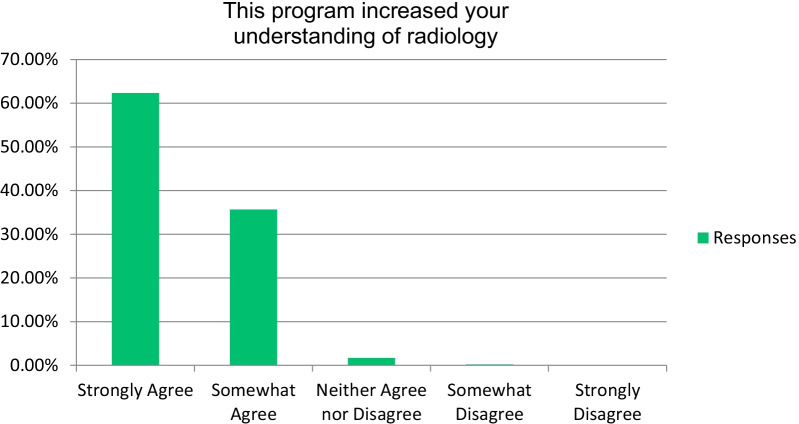
Respondents’ responses on the impact of the course on their radiology understanding

**Fig. 2:**
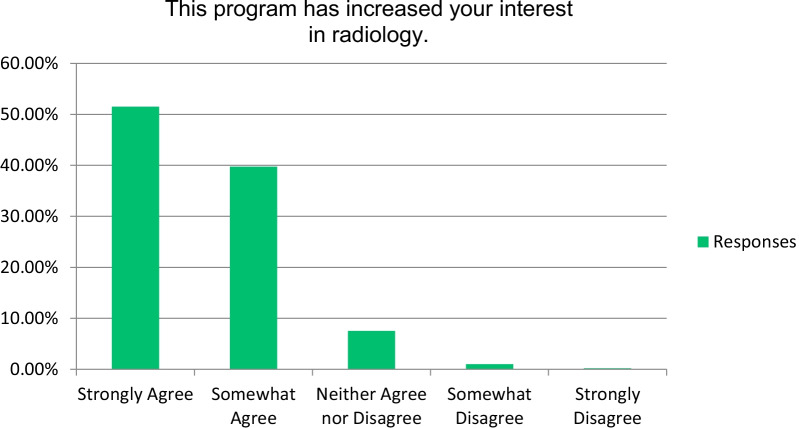
Respondents’ responses on the impact of the course on their interest in radiology

**Fig. 3:**
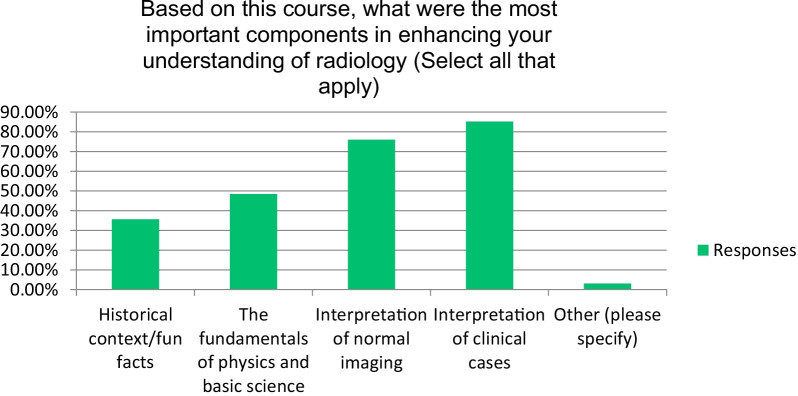
Percentage of respondents rating the most important factor to enhance radiology understanding

A total of over 97% of the attendees agreeing that the program increased their understanding of radiology and will be useful in their clinical practice in the future (Fig. 1). Also, more than 91% of the attendees agreed that the program increased their interest in radiology (Fig. 2). Most students (over 96%) strongly or somewhat agreed that the course was a worthwhile experience. Particularly, most respondents stated that among the most important components in enhancing their understanding of radiology were “the interpretation of normal imaging” and “interpretation of clinical cases” (Fig. 3). Seventy-nine percent of the respondents stated that “the amount of effort to complete the requirements for this program was just right.”

Students were asked to rate Phase I sessions on a 5-point Likert score. The average rating for all 9 sessions was 4.12 points, which translates to 80% of the sessions being rated good or excellent. Fifty-eight percent of participants reported that topics presented in the program were “excellent and clinically important to learn,” and 39% of participants reported that they were “good and somewhat important to learn.” More than 90% of students stated they are “strongly” or “very” likely to recommend this program to others.

**Fig. 4:**
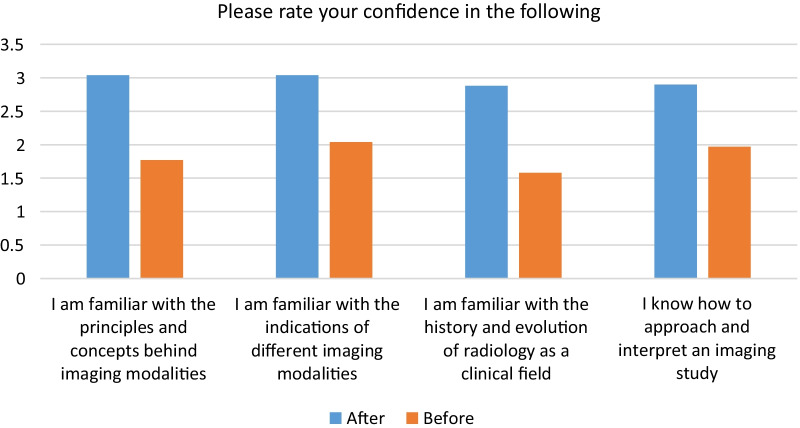
Comparison of the respondents’ confidence before and after the course

**Fig. 5:**
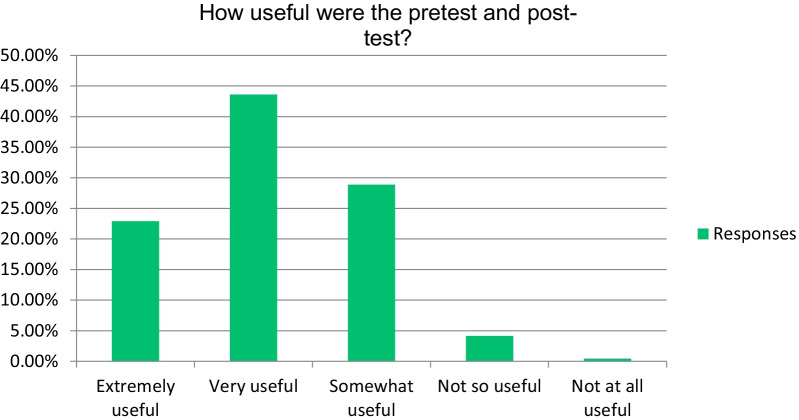
Respondents’ ratings on the usefulness of the pre- and post-tests

Subjective assessment of students’ confidence level was performed in the end-of-course survey to assess their confidence using and interpreting different imaging modalities. This assessment used a 4-point Likert score: not confident at all = 1 point, somewhat confident = 2, moderately confident = 3, and very confident = 4 (Fig. 4). Statistical significance (*p* < 0.001) was achieved regarding their confidence level before and after the course (Table 2). Subjective data regarding usefulness of both the pre-test–post-test model were measured (Fig. 5).

## Discussion

COVID-19 made a paradigm shift in undergraduate medical education in many ways [4, 17, 18]. This shift affected in-person interactive education and led to searching for alternatives, such as virtual learning and classroom, even in Egypt [7, 19]. The number of medical education webinars has drastically increased since then [20, 21]. With the help of online social media, virtual learning was introduced as an alternative tool and caused a shift from face-to-face interaction to online webinars. Zoom is one of the most used platforms for medical education today [5, 22].

Despite the rising importance of radiological imaging, radiology teaching continues to be fragmented globally [23–27]. One study showed lack of radiology education infrastructure in 13 African countries, including Egypt [14]. Therefore, we implemented a virtual radiology education model for medical students in Egypt. Our model was divided into two phases: phase I of 9 pre-recorded lectures, and phase II of 5 live sessions. We conducted phases I and II sessions via YouTube (YouTube, Inc., San Bruno, CA) then Zoom, respectively. From the registration forms, nearly half (45%) of participants stated that a radiology clerkship was not required at their medical school. This might have contributed to high attendance rate of all sessions (83%), with an average session rating of 4.12 out of 5. We used a pre-test–post-test design to assess students’ performance before and after each session; we achieved statistical significance for all sessions after comparing between pre- and post-test results. Several subjective data were assessed before and after the course regarding students’ level of confidence and knowledge in interpretation of different radiographs, familiarity of best-use radiological practices, and the utility of variable radiological modalities (Table 2).

In the end-of-course survey, 62% and 51% of students strongly agreed that the course increased their understanding and interest of radiology, respectively (Figs. 1, 2). Most participants (72%) considered radiology as a future career at the course end-survey. Therefore, we believe our course matched students’ knowledge level about radiology which is evidenced by the vast majority (79%) of students evaluating the course content to be “just right” and clinically important. Similar studies were achieved in the United Kingdom, India, and Australia regarding students’ perception of interventional radiology as a specialty [23, 24, 26].

Overall, we suggest from our study that increased exposure to radiology would augment students’ desire to pursue radiology as a future career. We also emphasize the role of social media platforms in introducing online courses to medical students. Since this course was designed solely for medical students, we wish to introduce a more specialized course for radiology residents. We hope this course will highlight radiology as a separate specialty as well, not a minority compared to other specialties.

## Limitations

A limitation of this study was that there was small participation of medical graduates (5%) into the course despite being designed exclusively for medical students. The registration form did not include a question inquiring the candidates about the name of their medical school in Egypt (which we will add to future registration forms); only the country of medical school was asked. Therefore, in ambassadors’ recruitment, we tried to cover most of Egyptian medical schools. Another limitation was the participation of a very tiny portion from other countries (0.86%). In addition, topics regarding safety and radiation hazards were not discussed separately in our course, but rather with other topics. The course was mainly designed to teach medical students about basics of radiology as a specialty and how to interpret the different imaging modalities and when to order them. We hope to address the issue of radiation hazards and how to prevent them in a standalone session, and we aim to address all these limitations in our future cohorts when we apply it on a larger scale.

## Conclusions

An online course of teaching fundamentals of imaging modalities could be delivered through an online format to medical students in a single or several countries and could be recorded to be accessible anytime by them. Focusing on a single modality of imaging at a time helps students learn radiology fundamentals, interpret studies, and locate abnormalities.

Nearly half of Egyptian medical students who participated in our cohort believe that imaging interpretation is under-taught in their curricula. Students eagerly participated and helped in the implementation of the virtual course, overcoming COVID-19 restrictions and increasing their knowledge and interest in radiology as a specialty.

A precise understanding of radiology principles is imperative to enhance clinical decision-making and their proper use in medicine. Thus, a multidisciplinary approach following radiology fundamentals can be complementary to the course and enrich students’ perception of radiology learning through filling possible gaps in undergraduate radiology education.

## Data Availability

Not applicable.

## References

[1] Murphy B (2020) COVID-19: how the virus is impacting medical schools. Am Med Assoc

[2] Sadek G, Kora M (2020). Transformation to virtual training during COVID-19 pandemic: case report from a low resources’ country. J Microsc Ultrastruct.

[3] Shehata MH (2020). Medical education adaptations post COVID-19: an Egyptian reflection. J Med Educ Curric Dev.

[4] Goh P, Sandars J (2020). A vision of the use of technology in medical education after the COVID-19 pandemic. MedEdPublish.

[5] Joia LA, Lorenzo M (2021). Zoom in, zoom out: the impact of the covid-19 pandemic in the classroom. Sustainability.

[6] Ismail II, Abdelkarim A, Al-Hashel JY (2021). Physicians’ attitude towards webinars and online education amid COVID-19 pandemic: when less is more. PLoS ONE.

[7] Abbas AM (2020). COVID-19 pandemic and medical education in a developing country. Am J Biomed Sci Res.

[8] Elsayes KM (2021). Online liver imaging course; pivoting to transform radiology education during the SARS-coV-2 pandemic. Acad Radiol.

[9] Belfi LM, Dean KE, Bartolotta RJ, Shih G, Min RJ (2021). Medical student education in the time of COVID-19: a virtual solution to the introductory radiology elective. Clin Imaging.

[10] Gunderman RB, Siddiqui AR, Heitkamp DE, Kipfer HD (2003). The vital role of radiology in the medical school curriculum. Am J Roentgenol.

[11] Straus CM (2014). Medical student radiology education: summary and recommendations from a national survey of medical school and radiology department leadership. J Am Coll Radiol.

[12] Schiller PT, Phillips AW, Straus CM (2018). Radiology education in medical school and residency: the views and needs of program directors. Acad Radiol.

[13] Dmytriw AA, Mok PS, Gorelik N, Kavanaugh J, Brown P (2015). Radiology in the undergraduate medical curriculum: too little, too late?. Med Sci Educ.

[14] Rehani B (2017). Radiology education in Africa: analysis of results from 13 African countries. J Am Coll Radiol.

[15] Frehywot S (2013). E-learning in medical education in resource constrained low- and middle-income countries. Hum Resour Health.

[16] Nabhani Y (2021). Multidisciplinary approach of teaching radiology to medical students in Egypt : is this an effective method?. Egypt J Radiol Nucl Med.

[17] El-Monshed AH, El-Adl AA, Ali AS, Loutfy A (2021) University students under lockdown, the psychosocial effects and coping strategies during COVID-19 pandemic: a cross sectional study in Egypt. J Am Coll Health 1–12 10.1080/07448481.2021.1891086 33651672

[18] Lucey CR, Johnston SC (2020). The transformational effects of COVID-19 on medical education. JAMA.

[19] Durfee SM (2020). Medical student education roadblock due to COVID-19: virtual radiology core clerkship to the rescue. Acad Radiol.

[20] Ahmed H, Allaf M, Elghazaly H (2020). COVID-19 and medical education. Lancet Infect Dis.

[21] Ismail II, Abdelkarim A, Al-Hashel JY (2021). Physicians’ attitude towards webinars and online education amid COVID-19 pandemic: when less is more. PLoS ONE.

[22] Serhan D (2020). Transitioning from face-to-face to remote learning: students’ attitudes and perceptions of using Zoom during COVID-19 pandemic. Int J Technol Educ Sci.

[23] Muzumdar S (2019). Understanding the awareness, knowledge and perceptions of interventional radiology amongst undergraduates in the UK. Cardiovasc Intervent Radiol.

[24] Foo M (2018). Australian students’ perspective on interventional radiology education: a prospective cross-institutional study. J Med Imaging Radiat Oncol.

[25] Emin EI (2019). Are interventional radiology and allied specialities neglected in undergraduate medical education? A systematic review. Ann Med Surg.

[26] O’Malley L, Athreya S (2012). Awareness and level of knowledge of interventional radiology among medical students at a Canadian institution. Acad Radiol.

[27] Alchallah MO (2020). Assessing diagnostic radiology knowledge among Syrian medical undergraduates. Insights Imaging.

